# The impact of common language on international trade: Evidence from Korean language

**DOI:** 10.1371/journal.pone.0307914

**Published:** 2024-08-15

**Authors:** Qingshu Xia, Boxu Zhou

**Affiliations:** 1 School of International Business, Southwestern University of Finance and Economics, Chengdu, China; 2 School of Finance and Management, Sichuan University of Arts and Science, Dazhou, China; University of International Business and Economics, CHINA

## Abstract

This study investigates the impact of a minority language on international trade. Using the distance to Hunchun as an instrumental variable, and transaction-level customs data from 2000 to 2015, we investigate the causal impact of common Korean language on international trade between Chinese counties and South/North Korea. The results suggest that a 1% increase in the proportion of population speaking Korean will increase value share and transactions share in trade by 1.8% and 2.3%, respectively. These effects are more pronounced in trade with North Korea and in higher trade share regions. Furthermore, we show that the common Korean language exerts its influence through reducing communication barrier. The impact is mainly observed at the extensive margin rather than the intensive margin. These findings highlight the potential of leveraging minority languages to boost bilateral trade in developing countries.

## 1. Introduction

New evidences have suggested that common language plays a crucial role as a bridge of communication in international trade [1–3]. The role of common language serves as an essential facilitator for economic transactions and relationship-building. Language is not merely a medium for communication, but also a crucial infrastructure that underpins the efficiency and cost-effectiveness of trade activities. For instances, common language reduces informational asymmetry, mitigates risks of opportunistic behavior, and minimizes negotiation and contracting costs. These savings can be substantial and often manifest in the form of reduced time lags and better pricing mechanisms. Existing studies have explored the impact of English, European languages, and Chinese on international trade, while the impact of other languages on trade has not been well sufficiently explored. Moreover, the evidence for developing countries is scarce. In this paper, we examine the impact of Korean language, spoken by about 78 million people across two countries around the world. Answering this question is a key to determine the role of common language, especially minority language, in economic development of developing countries.

Based on county-level import and export trade data from China for the period from 2000 to 2015, this paper explores whether the common Korean language (CKL) affects the trade between China and South/North Korea. We use the proportion of the Korean population to measure common Korean language. By employing the distance to Hunchun as an instrument defended by the unique historical Korean immigration at the end of the Qing Dynasty of China, we investigate the influence of CKL on bilateral trade.

We find as follows: Firstly, a 1% increase in the share of CKL leads to an increase in the value share and transactions share by 1.8% and 2.3%, respectively. It suggests a large elasticity of trade to CKL. Secondly, the impact is more pronounced in North Korea as compared to South Korea, highlighting nuanced variances possibly due to geopolitical or cultural differences. Thirdly, regions with higher proportions of trade are more significantly influenced by CKL, potentially pointing to the existence of economies of scale or network effects in these areas. Fourthly, CKL significantly reduces the price of the same commodity, implying reducing communication barrier. Fifthly, the common language is significantly correlated with a wider diversity in the categories of goods, methods of trade, and modes of transportation, demonstrating that common language can enrich the complexity and adaptability of bilateral trade relations. Lastly, the effects are predominantly evident in the quantity per transaction rather than in the value per transaction, suggesting that the extensive margin is more affected.

The marginal contributions of our study are as follows: Firstly, this paper contributes to the existing body of literature on the causal impact of common language on international trade by employing distance to Hunchun as an instrument, defended by the historical Korean immigration. Secondly, the paper delves into transaction-level impacts by employing a novel customs dataset spanning 16 years. This allows for a more nuanced understanding of how common language influences trade on a transaction-by-transaction basis. Thirdly, the paper provides empirical evidence to substantiate the theoretical proposition that common language acts as an effective tool in international trade, thereby confirming its economic utility. These contributions collectively enrich both the empirical and theoretical understanding of the economic functions of common languages in the context of international trade.

The remainder of the paper is organized as follows. Section 2 is literature review. Section 3 introduces the data sources and empirical strategy. Section 4 reports the empirical results including baseline results, the validity of instrumental variable (IV), heterogeneity, and additional robustness checks. Section 5 explores the mechanisms. Finally, Section 6 concludes.

## 2. Literature review

Language serves as an instrumental medium that influences cultural identity and even has palpable ramifications on economic and social behaviors, as highlighted by Ginsburgh and Weber [2]. The linguist Greenberg [4] was among the first to propose a potential link between language and economics, suggesting the incorporation of linguistic distance as a diversity index into research frameworks that explore political, economic, geographical, historical, and other non-linguistic factors. This idea has been subsequently cited by various scholars, pivoting the focus toward the intricate relationship between language and economy, including international trade, migration, translational communication, commerce, foreign investment and financial transactions [1–3,5–11]. Common language, serving as a conduit for communication, plays a pivotal role in social science research, particularly in the field of international economics, which is already enriched by a plethora of theoretical studies [12–15]. Therefore, this study contributes to this strand of literature illuminating the multifaceted implications of common language in international trade.

In empirical research, more attention has been given to the role of language as a non-tariff trade barrier in international trade [3,16,17]. Existing studies have noted the impact of linguistic diversity, such as dialects, on foreign direct investment and domestic regional trade [6,18–20]. While some studies may not explicitly draw a direct connection between language and trade, they nonetheless underscore that language can mitigate transaction costs associated with communication, as seen in contexts such as immigration [21,22] and cultural identification [23,24].

However, most existing empirical studies face challenges in identifying the causal impact of common language on international trade. In fact, some confounding factors such as economic development, culture, and institutional background may also affect trade. Egger and Lassmann [25] conducted a meta-analysis based on 81 studies examining the influence of language on trade. They find that the estimates were highly sensitive to sample selection and the choice of control variables, suggesting the presence of endogeneity issues. Ginsburgh and Weber [2] in their literature review on the economics of language underscore that the importance of accurately identifying causality remains further attention. For example, Egger and Lassmann [1] ingeniously exploited the distinct linguistic distribution in Switzerland by utilizing a spatial regression discontinuity design to examine the causal effect of common language on international trade. Fidrmuc and Fidrmuc [9] exploit historical communist countries’ education systems as a natural experiment, and use the variation in foreign language skills between Western and Eastern countries to identify the causal impact of language on trade. Therefore, research on the causal impact of common language on trade remains relatively sparse.

Similarly, this paper leverages the distance to Hunchun as an IV, defended by the historical migration of the Korean ethnic group to the three northeastern provinces of China. By doing so, we use the IV approach to identify the causal relationship between the CKL and contemporary trade between China and South/North Korea (including both South and North Korea).

## 3. Data and empirical strategy

### 3.1. Data

This empirical study mainly relies on China transaction-level customs data from 2000 to 2015, China ethnic population census data in 2000 and Korean migration history.

The transaction-level import/export data originate from RESSET customs database (http://www.resset.cn/enicip). This database provides detailed records of each import and export transaction. Variables include commodity code (HS8), trade value (in USD), quantity, trading modes, transportation mode, company name, address, and postal code. The chosen time frame from 2000 to 2015 is motivated by China’s accession to the WTO in 2001, a period when trade barriers were comparatively lower, thus alleviating endogeneity problems that other trade shocks may result in. Using information on addresses, postal codes, and enterprise names, we identify the county in which each company is located. Subsequently, following the methodology of Egger and Lassmann [1], we collapse this information at the county and country-to-origin/destination-country level across all years. Then, we calculate the *value share*, the ratio of each county’s aggregate value of transactions per country-to-origin or destination-country to its overall value of transactions for all countries; *transactions share*, the ratio of each county’s aggregate number of transactions per country-to-origin or destination-country to its overall number of transactions for all countries. To check the mechanisms, we also calculate the price of the same commodity, number of commodity classifications, number of trade modes, number of transportation modes, the value per transaction, quantity per transaction. In addition, we also calculate the total export and import value in each section of HS commodities (https://www.wcoomd.org) across counties, to explore how CKL affects trade structure.

To measure the common language shared by both China and South/North Korea, we use the 2000 China ethnic population census data [26]. The data document population for each ethnic group in each county. The share of Korean ethnic group relative to all population for each county is considered as the proficiency of common Korean language (CKL). Individuals belonging to a certain ethnic group are more likely to speak the language of their ethnicity. Thus, a higher share of Korean ethnic individuals in a specific area suggests that Korean is more likely to be spoken there, serving as a proxy for CKL proficiency. The Korean ethnic group in China possesses bilingual proficiency in both Mandarin and Korean, conferring upon them a competitive advantage in international trade. Specially, given that the majority of the ethnic Korean population resides in Northeastern China, we restrict the sample to three northeastern provinces, i.e. Jilin, Liaoning, and Heilongjiang. To address the potential issues related to the dimension mismatch, we utilize the 2010 China ethnic population census data, as the population census is conducted decennially. In addition, we also use the ratio of Korean-teaching schools as another proxy for CKL, which is obtained from Point of Interest (POI) data provided by Baidu map, one of the most widely used mapping and navigation applications in China. The data collection of POI is limited to the period from 2012 to 2015 due to data availability.

To alleviate the endogeneity problems, we construct an IV, i.e. the distance to Hunchun where the Korean mass migration happened in late Qing Dynasty of China. In early Qing, a large of areas in Northeast China bordering Korea were sealed off, limiting any large-scale population inflows [27]. However, in late Qing, as the government faced decline following the turmoil of the Opium War and Taiping Rebellion, it was unable to control the influx of refugees into the Sino-Korean border regions due to famine and war [28]. In 1881, the Qing government established a reclamation bureau in Hunchun and designated the area north of the Tumen River as a special reclamation zone for Korean immigrants [29]. This policy facilitated large-scale migration and led to Hunchun becoming an initial foothold for Korean immigrants. Over time, this developed into the Korean ethnic enclaves represented today by Yanbian Prefecture. Thus, the demographic distribution of ethnic Koreans in Northeast China has profound historical roots, reflecting the legacy of history and culture to a significant extent. As Falck et al. [6] noted in their research on the relationship between migration and language, massive migration flows lead to greater linguistic and cultural similarity in geographically proximate regions. Therefore, the distance to Hunchun can serve as a determinant of the extent to which various areas are influenced by Korean language. Closer proximity to Hunchun correlates with a greater influence from Korean culture and a higher number of Korean speakers. Accordingly, we use ArcGIS software to calculate the great circle distances from each county to Hunchun.

We also collect some control variables that may confound the analysis. First, we consider transportation amenities affecting trade with Korea. This category includes the distance of each county to the nearest coastline, the nearest railway in 1999, the nearest highway in 1999, and the nearest international airport in 2000. Second, geographical factors are used to control for resource endowment that may also affect trade. These variables are average elevation, terrain ruggedness, and agricultural suitability. Third, we control for initial socio-economic indicators including population density in 2000 and illiterate rate. We also control for nighttime luminosity in 2000 as initial economic development level, due to the unavailability of GDP for all counties in 2000. The data sources are in Table 1.

**Table 1:** Summary statistics. The data sources: A. RESSET Customs Data; B. Tabulation on nationalities of population census of China in 2000 and 2010 [26,30]; C. Calculation using ArcGIS; D. Baum-Snow et al. [31]; E. Authors’ collection; F. SRTM30 (http://www.webgis.com); G. Nunn et al. [32]; H. Ramankutty et al. [33]; I. China population census in 2000 (https://www.stats.gov.cn); J. DMSP-OLS nighttime luminosity in 2000 (https://ngdc.noaa.gov); K. Provincial capital cities: Shenyang, Changchun, and Harbin; L. POI (2012–2015) from Baidu map.

Variables	Mean	SD	Min	Max	N	Source
*Panel A*: *County-level data*						
Value share	14.12	22.63	0	100	4608	A
Transactions share	16.68	24.04	0	100	4608	A
Number of products (HS2)	14.32	21.09	0	94	4608	A
Number of trade modes	1.59	1.74	0	12	4608	A
Number of transportation modes	1.7	1.6	0	6	4608	A
Value per transaction (log)	8.09	5.16	0	17.74	4608	A
Quantity per transaction (log)	7.89	5.24	0	18.37	4608	A
Value share (with Japan)	12.29	20.15	0	100	4608	A
Transactions share (with Japan)	12.14	18.42	0	100	4608	A
Korean ethnic group ratio (CKL)	2.05	7.24	0.01	65.43	288	B
Distance to Hunchun	557.75	205.23	0	1237.08	288	C
Distance to nearest coastline	490.1	346.13	0.42	1315.72	288	C
Distance to nearest railway	15.27	15.8	0	86.94	288	C, D
Distance to nearest highway	84.05	94.44	0.34	709.61	288	C, D
Distance to nearest international airport	155.7	112.32	2.79	625.01	288	C, E
Elevation	245.45	184.42	0.81	1088.81	288	C, F
Ruggedness	7.59	6.27	0.19	27.55	288	C, G
Agricultural suitability	0.82	0.17	0.09	0.99	288	C, H
Population density (log)	5.55	1.72	0.62	10.39	288	I
Illiterate rate	6.23	2.32	0.87	14.75	288	I
Average nighttime luminosity (log)	1.33	1.48	-2.1	4.14	288	C, J
Provincial capital city	0.14	0.35	0	1	288	K
Ratio of Korean-teaching schools	0.53	2.3	0	36.84	1152	L
CKL including 2010	1.96	7.03	0	65.43	4608	B
Price of the same commodity	2.09	1.98	0	19.07	1916875	A
*Panel B*: *Prefecture-level data*						
Value share	6.39	9.17	0	100	5536	A
Transactions share	6.17	10.2	0	100	5536	A
CKL	0.21	1.99	0	36.26	346	B
Distance to Hunchun	2138.7	883.1	146.29	4481.9	346	C
Distance to nearest coastline	642.19	667.3	1.6	3500.93	346	C
Distance to nearest railway	53.49	117.79	0.02	1074.56	346	C, D
Distance to nearest highway	130.81	187.7	0.29	1419.99	346	C, D
Distance to nearest international airport	330.45	243.08	12.98	1616.27	346	C, E
Elevation	788.94	1015.76	1.05	5038.94	346	C, F
Ruggedness	158.25	128.56	3.46	765.26	346	C, G
Agricultural suitability	0.65	0.28	0	0.98	346	C, H
Population density (log)	5.27	1.5	-1.36	8.34	346	I
Illiterate rate	11.13	9.31	1.24	66.71	346	I
Average nighttime luminosity (log)	0.49	1.67	-6.85	3.93	346	C, J
Provincial capital city	0.09	0.29	0	1	346	K

### 3.2. Empirical strategy

This study utilizes the various proportions of the Korean ethnic population across counties in three northeastern provinces of China as a measure to gauge Korean language proficiency. To examine the impact of common Korean language on bilateral trade between Korea and China, we employ the customs data in 288 counties from 2000 to 2015. We start the Ordinary Least Squares (OLS) model as follows:

$$tradeShare_{ctp}=\beta CKL_{cp}+X_{c}\tau +\theta_{p}t+u_{p}+v_{t}+\varepsilon_{ctp}$$ (1)

where, following Egger and Lassmann (2015), *tradeShare*_*ctp*_ represents the outcome of interest: value share and transactions share with Korea for county *c* of province *p* in year *t*. *CKL*_*cp*_ is common Korean language, measured by Korean ethnic group ratio. ***X***_***c***_ is a vector of control variables including distance to nearest coastline/railway/highway/international airport, elevation, ruggedness, agricultural suitability, population density, illiterate rate, average nighttime luminosity, provincial capital city. Province-specific time trends are included as ***Q***_***p***_*t* to control for the differential development in different areas. This captures any idiosyncratic temporal variations that might exist within each province. *u*_*p*_ and *v*_*t*_ are province and year fixed effects. Province fixed effects control for unobserved factors that are specific to each province but remain constant over time, such as province-specific trade liberalization policies. Year fixed effects control for unobserved time-specific factors that may influence the outcome variable across different years but remain constant over counties, such as nationwide development trends and policy shifts. Thus, our identification strategy relies on within-province comparisons among counties with varying CKL and trade with South/North Korea, as well as on within-year comparisons among these counties. *ε*_*ctp*_ is the error term.

However, there are some endogeneity problems. Firstly, a potential reverse causality issue exists. While an increase in the Korean ethnic group may spur trade with Korea, trade activities could also make regions with higher trading volumes with Korea more attractive for Korean ethnic group to reside in. Secondly, some omitted variables are unobservable, such as more complex economic, cultural, and institutional factors, thus leading to a biased estimator. As noted by Egger and Lassmann [25] in their meta-analysis concerning the impact of language on international trade, the estimation results are highly sensitive to sample selection and the choice of control variables.

To address these endogeneity concerns, we use IV method. The IV is the shortest distance of each county to Hunchun where a reclamation bureau was established since the late Qing Dynasty of China, as discussed above in data sources. It enables us to employ the two-stage least squares (2SLS) method to identify the causal impact of CKL on bilateral trade. This method not only allows us to mitigate potential endogeneity issues, but also enables us to explore external policy instruments that can indirectly influence international trade by directly affecting the common language. While this method provides valuable insights, it is important to interpret the findings with caution regarding claims of causality. Thus, the first-stage equation estimates the impact of distance to Hunchun on the CKL:

$$CKL_{cp}=\delta d2Hunchun_{cp}+X_{c}\tilde{\tau }+u_{p}+\xi_{ctp}$$ (2)

where *d*2*Hunchun*_*cp*_ is the shortest distance to Hunchun (IV). Other variables are the same as Eq (1).

## 4. Empirical results

### 4.1. Baseline results

The OLS and 2SLS results are in Panel A and Panel B of Table 2, respectively. Columns (1)-(6) of both panels suggest that the coefficients are statistically significant at the 1% level, even after controlling for various confounding factors. Columns (3) and (6) in Panel A show that a 1% increase in CKL would increase value share and transactions share by 1% and 1.2%, respectively. However, OLS results hardly explain causality. Columns (3) and (6) in Panel B show the 2SLS results. A 1% increase in CKL increases value share and transactions share by 1.8% and 2.3%, respectively. These effects correspond to 13% of the mean value share and 14% of the mean transactions share, respectively, indicating the economic scale is large. Alternatively, a single standard deviation increase in CKL results in 13 (= 1.8*7.24) more percentage points in value share and 16.7 (= 2.3*7.24) more percentage points in transactions share, respectively. All the Kleibergen-Paap F-statistics are above 10, indicating no weak instrument. The inclusion of province-specific trend in Columns (3) and (6) does not significantly influence the coefficients, indicating that there are no substantial trends within the provinces. Hence, CKL has a causal impact on bilateral trade with South/North Korea.

**Table 2:** The impact of CKL on bilateral trade.

	Value share				Transactions share		
	(1)	(2)	(3)		(4)	(5)	(6)
*Panel A*: *OLS*							
CKL	1.004*** (0.087)	1.007*** (0.115)	1.007*** (0.115)		1.250*** (0.102)	1.239*** (0.131)	1.239*** (0.131)
Province and year FE	Yes	Yes	Yes		Yes	Yes	Yes
All controls		Yes	Yes			Yes	Yes
Province trend			Yes				Yes
R^2^	0.175	0.195	0.196		0.250	0.285	0.293
Observations	4608	4608	4608		4608	4608	4608
*Panel B*: *2SLS*							
CKL	1.115*** (0.269)	1.800*** (0.413)	1.800*** (0.413)		1.467*** (0.276)	2.254*** (0.466)	2.254*** (0.466)
Kleibergen-Paap F	14.6	23.1	23.1		14.6	23.1	23.1
Province and year FE	Yes	Yes	Yes		Yes	Yes	Yes
All controls		Yes	Yes			Yes	Yes
Province trend			Yes				Yes
Observations	4608	4608	4608		4608	4608	4608
*Panel C*: *Reduced-form*							
Distance to Hunchun	-0.017*** (0.006)	-0.044*** (0.009)	-0.044*** (0.009)		-0.023*** (0.007)	-0.056*** (0.009)	-0.056*** (0.009)
Province and year FE	Yes	Yes	Yes		Yes	Yes	Yes
All controls		Yes	Yes			Yes	Yes
Province trend			Yes				Yes
R^2^	0.099	0.171	0.173		0.148	0.255	0.263
Observations	4608	4608	4608		4608	4608	4608

Standard errors clustered at the county level in parentheses.

* p < 0.1

** p < 0.05

*** p < 0.01. All control variables include distance to nearest coastline, railway, highway, and international airport, elevation, ruggedness, agricultural suitability, population density (log), illiterate rate, average nighttime luminosity (log), provincial capital city. The same notes apply to subsequent tables.

The OLS estimates underestimate the 2SLS estimates. The downward bias of the OLS estimates could be attributable to counties that are active in international trade but have a relatively a small proportion of Korean ethnic group, such as Dalian city. Alternatively, the Korean ethnic group may have leveraged China’s WTO accession as an opportunity to migrate to counties where they can trade with Korea. Overall, various factors could attenuate the influence of the Korean ethnic group share. Consequently, without an appropriate IV, the impact of CKL on bilateral trade is likely to be confounded by some unobservable factors.

In addition, panel C of Table 2 shows the reduced-form results for the overall impact of the distance to Hunchun on trade with Korea. The distance to Hunchun exerts a significant influence on value share and transactions share. Columns (3) and (6) show that for every 100km closer to Hunchun, value share and transactions share will be increased by 4.4 percentage points and 5.6 percentage points, respectively. However, these reduced-form estimates may include both direct and indirect effects. If the direct effects are substantial, the validity of the 2SLS results could be compromised. We will discuss this in subsection 4.2.

### 4.2. The validity of IV

The validity of IV method requires two assumptions. The first one is the exclusion restriction, i.e. the distance to Hunchun affects trade with Korea by influencing only the proportion of the Korean ethnic group. The second one is the strong relationship between IV and Korean ethnic population ratio in the first stage.

#### 4.2.1. Exclusion restriction

**Fig 1:**
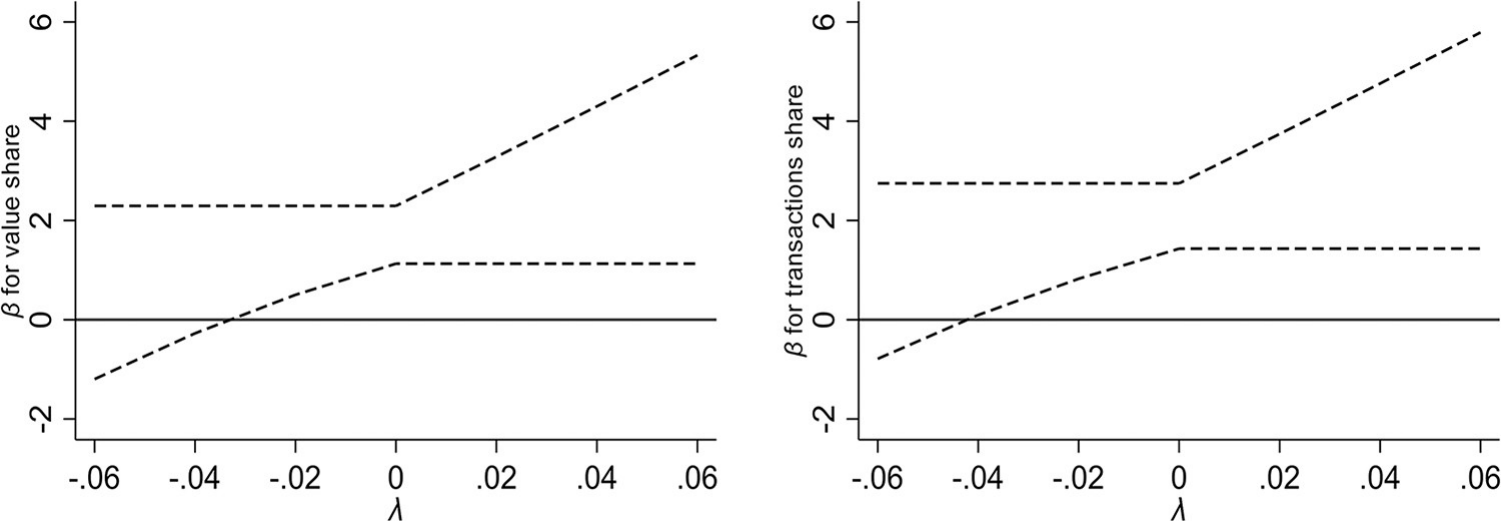
The impact of CKL on trade with plausibly exogenous IV.

To check the exclusion restriction of IV, we consider the plausibly exogenous approach which relaxes the assumption of strict exogeneity of IV [34]. This method is also applied in some empirical papers such as Calvi and Mantovanelli [35] and Guo [36]. This method puts the IV directly into the second-stage Eq (1) with coefficient *λ*. In this way, the distance to Hunchun is allowed to directly influence the trade with Korea. The coefficient of CKL reveals how the 2SLS estimation is influenced if *λ* takes on different values. Fig 1 shows the 2SLS estimation of CKL if the IV is plausibly exogenous with dashed lines indicating the 90% confidence intervals.

As Fig 1 reveals, when *λ* is positive, the estimates of CKL on trade with Korea remains always significantly positive. A positive sign on *λ* suggests that as the distance to Hunchun increases, the direct influence of CKL rises. For instance, Dalian as an international port city, despite its long distance to Hunchun, maintains a high volume of trade with South/North Korea. However, the 2SLS estimates remain significant and positive even if the distance to Hunchun directly affects trade.

When *λ* is negative, the estimates of CKL remain significantly positive as long as *λ* > −0.03. A negative sign on *λ* implies that when getting closer to Hunchun, the direct impact of CKL on trade with Korea rises. This result rules out a critical mechanism, i.e. the increase of trade with Korea mainly stems from shorter distance. In other words, in some counties near Hunchun, the impact of CKL persists. Although the coefficient of 2SLS is no longer significant when *λ* < −0.03, this threshold is as large as half of the overall reduced-form coefficients (-0.044 and -0.056) as reported in Panel C of Table 2. Thus, despite the fact that closer proximity to Hunchun is associated with more trade with Korea, the 2SLS estimation still confirms the significant impact of CKL on trade as long as the direct impact of distance to Hunchun on trade do not exceed one half of the overall reduced-form effect.

#### 4.2.2. First stage results

To check if the IV, namely the distance to Hunchun, is strong correlated with Korean ethnic group ratio. Table 3 reports the results when other control variables are included. It shows that the impacts of distance to Hunchun on CKL remain significant and negative, suggesting that counties with closer proximity to Hunchun are more likely to have a higher proportion of Korean ethnic group. Therefore, there is no weak IV.

**Table 3:** First stage results.

	Korean ethnic group ratio (CKL)				
	(1)	(2)	(3)	(4)	(6)
Distance to Hunchun	-0.014*** (0.002)	-0.021*** (0.002)	-0.026*** (0.003)	-0.026*** (0.003)	-0.025*** (0.003)
Transportation amenities		Yes	Yes	Yes	Yes
Geographical factors			Yes	Yes	Yes
Socio-economic factors				Yes	Yes
Province FE					Yes

### 4.3. Heterogeneous effects

Heterogeneity analysis is conducive to understanding different impacts of common language. We first check the multifaceted impacts of CKL on trade with North and South Korea, respectively. We then check the impacts of CKL on import and export. Additionally, we employ unconditional quantile regression to estimate across the distribution of trade shares.

**Fig 2:**
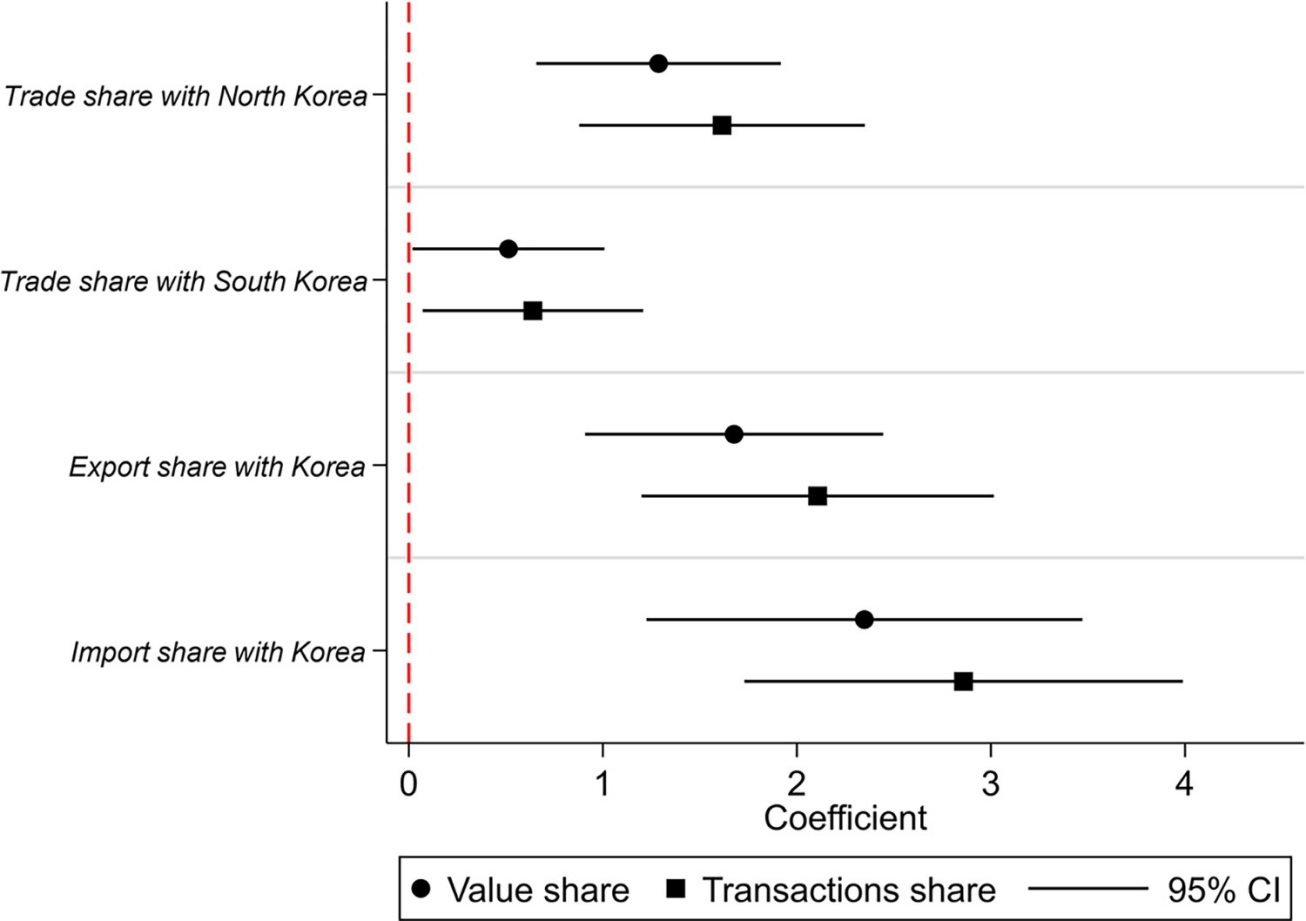
Heterogeneous effects of CKL on bilateral trade.

Firstly, we explore the heterogeneous impacts of CKL on trade with North and South Korea. As revealed in Fig 2, CKL exerts a significant positive influence on both North Korea and South Korea. Moreover, the impact on North Korea is greater than on South Korea.

Secondly, we check the impact on export and import share with Korea, respectively. CKL has a significant impact on both import and export, but the different between the coefficients are not significant.

**Fig 3:**
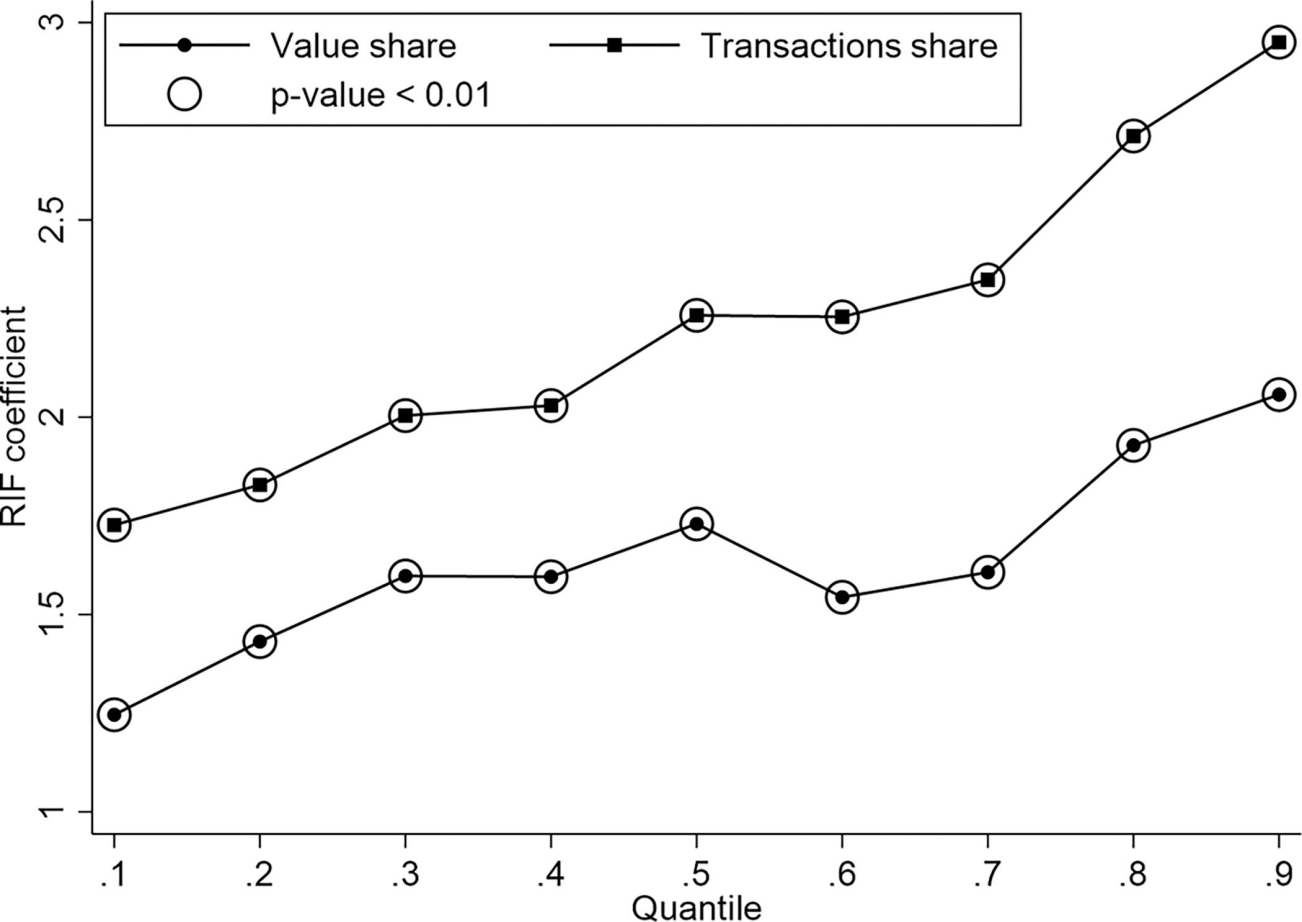
Unconditional quantile regression results.

Thirdly, Fig 3 reveals the quantile regression results. We employ the unconditional quantile regression (UQR) approach proposed by Firpo et al. [37]. While the traditional conditional quantile regression generates results that are sensitive to different sets of covariates, UQR provides more interpretable results irrespective of other covariates. The UQR employs Recentered Influence Function regression (RIF) to estimate the marginal effects of small changes in the Korean ethnic share at different trade share quantiles. Fig 3 separately presents the RIF coefficients for value share and transactions share, respectively. The marginal effects of CKL are more pronounced at higher quantiles than at lower ones for both value share and transactions share, implying a higher impact of CKL in regions with higher trade share. Therefore, the influence of CKL varies across regions with different trade shares, and regions with higher trade shares are more pronounced.

### 4.4. Robustness checks

In this subsection, we further check whether the results are robust in relation to alternative CKL, alternative IV, other provinces as a control group, excluding extreme cases, dynamic differences, and placebo tests.

#### 4.4.1. Alternative CKL

To address some drawbacks of CKL in baseline results, we conduct two sets of additional robustness checks. First, although we select Korean ethnic group ratio as a primary proxy for CKL, it may not entirely indicate the ability to communicate with Korea. Therefore, we consider the ratio of Korean-teaching schools as another proxy for CKL, as it can suggest the ability to communicate with South/North Korea to some degree. If the word “chao xian” (“Korean” in Chinese) appears in the name of an educational institution, this institution is considered as a Korean-teaching school. The share of Korean-teaching schools is calculated by dividing the number of Korean-teaching institutions by the total number of schools in a county. The results are reported in Table 4. All results show that the share of Korean-teaching schools also has significant impact on value share and transactions share. The first stage Kleibergen-Paap F-statistics in Column (1) and (4) are less than 10, indicating weak instrument. Hence, we also report the Anderson-Rubin confidence intervals. They are all significantly positive, showing valid 2SLS results.

**Table 4:** Alternative CKL: Ratio of Korean-teaching schools and CKL including 2010.

	Value share			Transactions share		
	(1)	(2)	(3)	(4)	(5)	(6)
Ratio of Korean-teaching schools	11.507**			13.398**		
	(5.298)			(6.013)		
CKL including 2010		1.867***	1.838***		2.338***	2.150***
		(0.430)	(0.471)		(0.488)	(0.518)
Kleibergen-Paap F	5.0	22.4	21.5	5.0	22.4	21.5
Anderson-Rubin CI	[5.0,. . .]	[1.2, 3.1]	[1.0, 3.2]	[6.5,. . .]	[1.6, 3.8]	[1.3, 3.6]
Province and year FE	Yes	Yes	Yes	Yes	Yes	Yes
All controls	Yes	Yes	Yes	Yes	Yes	Yes
Province trend	Yes	Yes	Yes	Yes	Yes	Yes
Observations	1152	4608	576	1152	4608	576

Second, we include Korean ethnic group ratio in 2010 to highlight the potential issues related to the dimension mismatch. The CKL in baseline results is obtained from census 2000, which is time-invariant. To ensure consistency across the dataset, we use the CKL in 2010 from census 2010. Specifically, the CKL in 2000 is applied to the period 2000–2009 while the CKL in 2010 is applied to the period 2010–2015. The results are reported in Column (2) and (5), they are still significant and consistent with baseline results. In Column (3) and (6), we limit the dataset in two years, i.e. 2000 and 2010. The results remain robust.

#### 4.4.2. Alternative IV

Since some counties far from Hunchun have almost no Korean ethnic population, there may not be a linear correlation between CKL and the distance to Hunchun. To address this, we use an alternative IV, the log-form of distance to Hunchun which can mitigate the impact of some outliers. Table 5 reports the results which show the significant impact of CKL, although the estimates are slightly lower than baselines. All Kleibergen-Paap F statistics are still greater than 10, showing the robustness results.

**Table 5:** Alternative IV: Log-form distance to Hunchun.

	Value share			Transactions share		
	(1)	(2)	(3)	(4)	(5)	(6)
CKL	0.944***	1.168***	1.168***	1.418***	1.784***	1.784***
	(0.134)	(0.204)	(0.204)	(0.149)	(0.231)	(0.231)
Kleibergen-Paap F	20.9	11.0	11.0	20.9	11.0	11.0
Province and year FE	Yes	Yes	Yes	Yes	Yes	Yes
All controls		Yes	Yes		Yes	Yes
Province trend			Yes			Yes
Observations	4608	4608	4608	4608	4608	4608

#### 4.4.3. Other provinces as a control group

**Fig 4:**
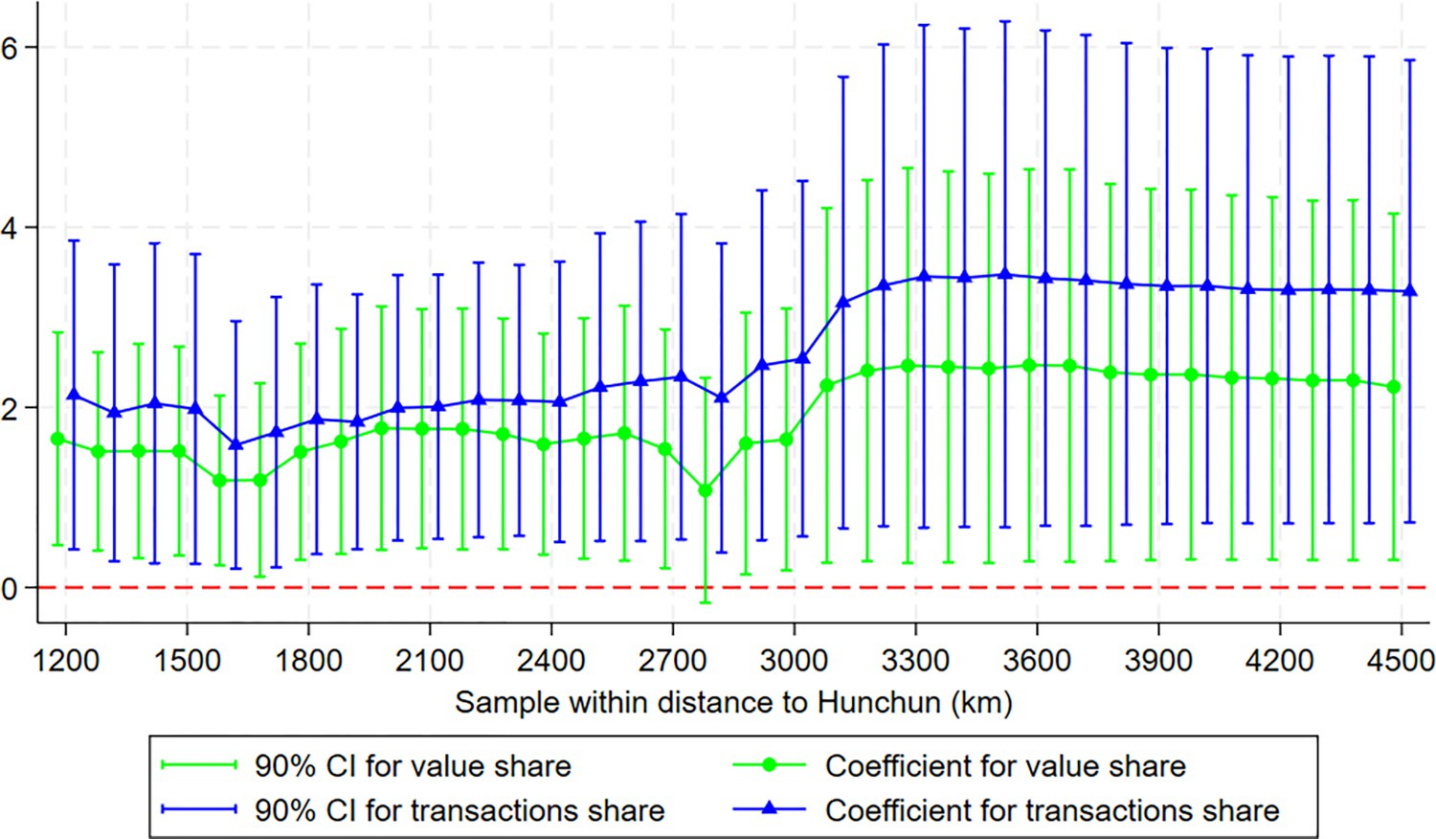
Results when increasing the sample within distance to Hunchun.

As the three Northeast provinces are closely related to North Korea, we include other provinces as a control group. For the sake of simplicity, we use Chinese prefecture-level cities instead of counties. As the distance from Hunchun increases, the share of Korean ethnic population tends to decrease, sometimes reaching zero. To ensure comparability and test the robustness of the results, we gradually include samples from areas farther away from Hunchun for the estimates. We start from 1200 km distance to Hunchun because this range just encompasses all three northeastern provinces. Fig 4 shows that the CKL still has impacts on both value share and transactions share.

#### 4.4.4. Excluding extreme cases

Table 6 reports results when dropping some extreme cases. Columns (1)-(2) show the results after excluding all counties in Dalian prefecture, which serves as a major coastal port in the Northeast China and could potentially lead to estimation bias. Columns (3)-(4) drop counties that had no any trade from 2000–2006, thereby removing the possible manipulation by counties that are inactive in international trade. Columns (5)-(6) remove counties within provincial capitals as the trade activities of these counties could be influenced by favorable policies. The results from columns (1)-(6) suggest that even after dropping these extreme cases, the coefficients for CKL remain significant and positive. Overall, the results are robust when excluding some extreme cases.

**Table 6:** Extreme cases.

	Dropping counties within Dalian		Dropping counties with no trade		Dropping counties within provincial capital cities	
	Value share	Transactions share	Value share	Transactions share	Value share	Transactions share
	(1)	(2)	(3)	(4)	(5)	(6)
CKL	1.720*** (0.418)	2.187*** (0.472)	1.806*** (0.414)	2.259*** (0.467)	1.849*** (0.433)	2.329*** (0.492)
Kleibergen-Paap F	23.7	23.7	23.1	23.1	22.9	22.9
Province and year FE	Yes	Yes	Yes	Yes	Yes	Yes
All controls	Yes	Yes	Yes	Yes	Yes	Yes
Province trend	Yes	Yes	Yes	Yes	Yes	Yes
Observations	4448	4448	4592	4592	3952	3952

#### 4.4.5. Dynamic effects

**Fig 5:**
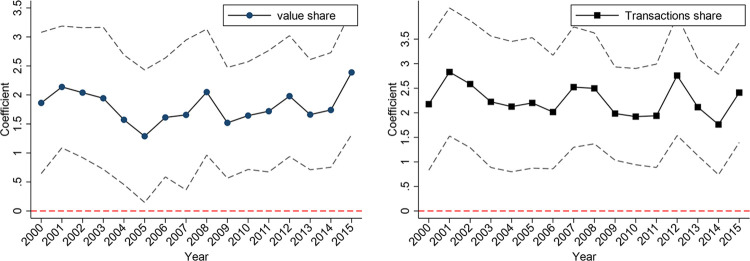
Dynamic effects of CKL on trade for each year.

In this subsection, we investigate whether the impact of CKL on trade changes over time by estimating the effects for subsamples of each year from 2000 to 2015. Fig 5 reports the dynamic effects for this period, with dashed lines representing the 95% confidence intervals. The results for each year are independently analyzed, meaning there is no reference group for comparison across different periods. The results show little variation and remain significant, confirming the robustness of the findings.

#### 4.4.6. Placebo tests

**Fig 6:**
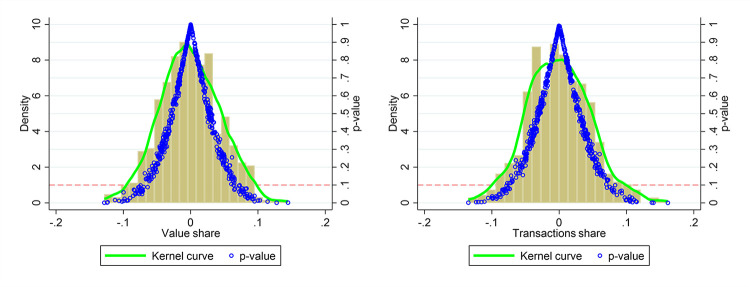
Placebo test I: Random assignment of CKL.

To check if our results are randomly generated, we conduct two placebo tests. Firstly, we randomly assign values of Korean ethnic share within the range [0.01, 65.43] to 288 counties. Then we use this sample to perform OLS regression in Eq (1). We repeat this process 500 times to obtain distributions of all coefficients and corresponding p-values. Due to the random selection of CKL treatment group, most coefficients should be insignificant and close to zero. Fig 6 reveals that these coefficients are clustered around zero. Only a small number of the coefficients are significant at the 10% level, but they are substantially smaller than the actual coefficients (1.007 and 1.239) reported in Table 2. This suggests that random assignment of CKL does not generate the baseline results, confirming the robustness of the findings.

**Fig 7:**
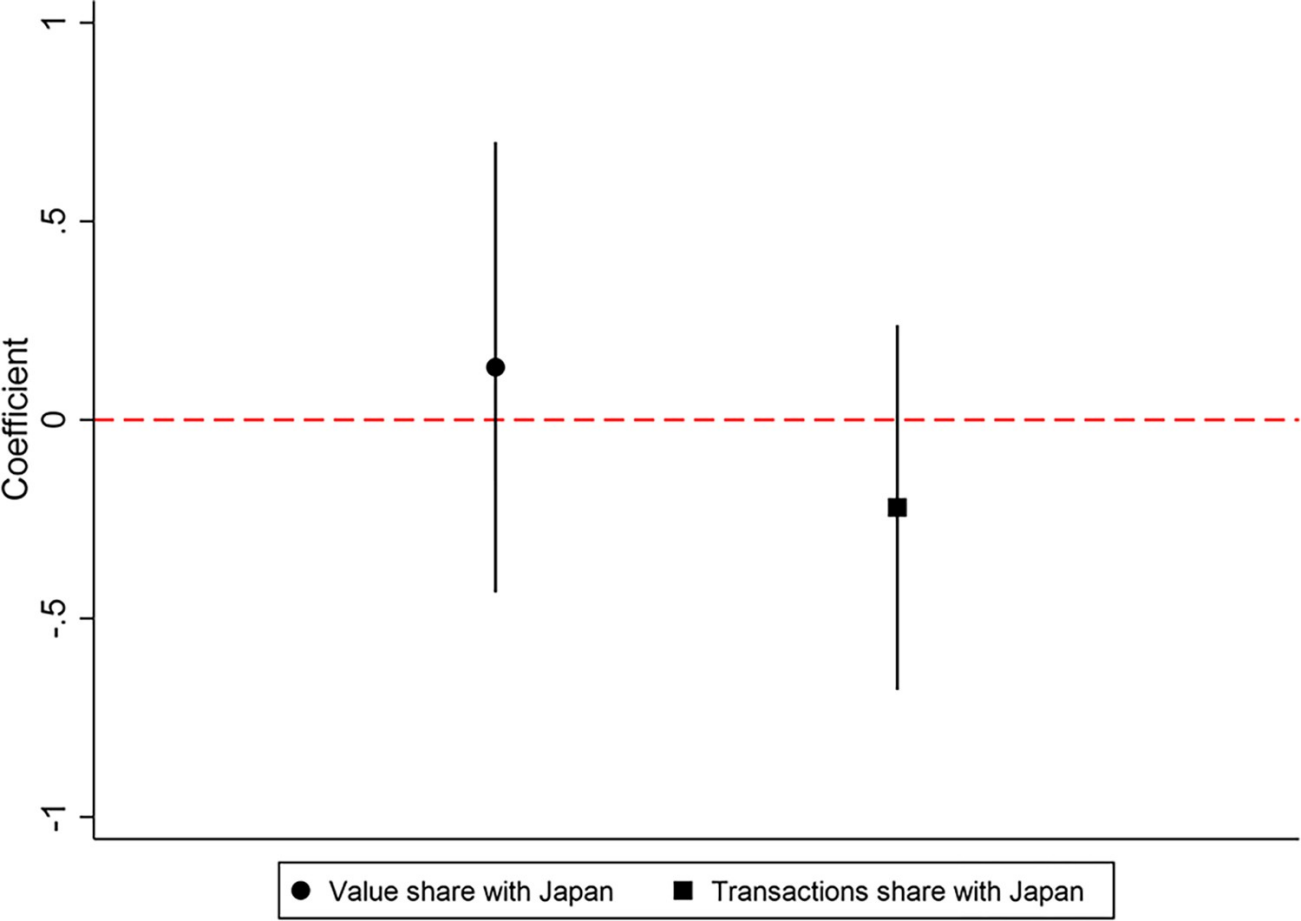
Placebo test II: The impact of CKL on trade with Japan.

Secondly, we check if the impact of CKL on trade with Japan. If CKL has a significant impact on trade share with other similar countries, the validity of our argument would be compromised. To address this concern, we test whether CKL has a significant impact on trade with Japan. We select Japan for two reasons: First, Japan is geographically close to China and shares some aspects of East Asian culture. If CKL has any impact on trade between China and Japan, it could undermine the reliability of the argument. Second, since China’s entry into the WTO, Japan has been another significant trading partner near China. Fig 7 shows the placebo test evaluating the impact of the CKL on trade with Japan. The findings reveal that neither value shares nor transactions share is affected by CKL. Thus, CKL only affects trade with countries that share a common language.

## 5. Mechanisms

In this section, we further explore why common language affects trade. Common language is more likely to reduce communication barrier, therefore we mainly check if the price of the same commodity, number of commodity classifications (HS2), number of trade modes, number of transportation modes, are driven by CKL. Then we check if the impact of CKL is at the intensive margin or extensive margin. Lastly, we also explore which section of HS commodities is affected by CKL to understand how CKL impacts the trade structure.

Table 7 shows all results indicating possible channels. Column (1) estimates the effect of CKL on price of the same commodity across counties. Because common language reduces information asymmetry, the market will be more competitive. Therefore, we would expect a lower price of the same commodity in counties with higher CKL. To eliminate variability due to differences in commodity classification and measurement, we include HS8-by-unit fixed effects, which ensures that comparisons are made within the same HS8 commodity and the same unit. The result shows that a 1% increase in CKL decreases the price of the same commodity by 1.5%. Thus, CKL is more likely to help reduce communication barrier, resulting in more competitive market.

**Table 7:** Mechanisms. Trade modes determines the customs clearance rate of traded goods, such as general trade, processing trade and compensatory trade, etc. Transportation modes include railways, mails, airlines, automobile, waterways, etc.

	Price of the same commodity (log)	Number of commodity classifications (HS2)	Number of trade modes	Number of transportation modes	Value per transaction (log)	Quantity per transaction (log)
	(1)	(2)	(3)	(4)	(5)	(6)
CKL	-0.015*** (0.004)	1.216** (0.507)	0.077*** (0.028)	0.065** (0.027)	0.114 (0.074)	0.154** (0.077)
Kleibergen-Paap F	82.2	23.1	23.1	23.1	23.1	23.1
Province and year FE	Yes	Yes	Yes	Yes	Yes	Yes
All controls	Yes	Yes	Yes	Yes	Yes	Yes
Province trend	Yes	Yes	Yes	Yes	Yes	Yes
HS8-by-unit FE	Yes					
Observations	1916875	4608	4608	4608	4608	4608

In addition, column (2) indicates that CKL significantly increases the number of commodity classifications (HS2), implying that a shared common language facilitates companies in discovering a wider range of products and exploring new markets. Column (3) shows that the number of trade modes is also significantly increased by CKL, suggesting that common language plays an essential role in customs procedures between China and South/North Korea. Column (4) reveals that CKL significantly increases the variety of transportation modes. Selecting appropriate transportation mode can reduce transaction costs. An increase in the variety of transport methods indicates that a shared common language also aids in reducing the costs associated with international trade logistics.

Following Egger and Lassmann [1], columns (5) and (6) check if the effect of CKL is at extensive margin or intensive margin. Column (5) shows that the impact of CKL on value per transaction is not significant, implying CKL does not matter for intensive margin. However, column (6) reveals a positive and significant effect on the quantity per transaction. These results align with Egger and Lassmann [1] who argue that common language only matters for extensive margin rather than intensive margin.

**Fig 8:**
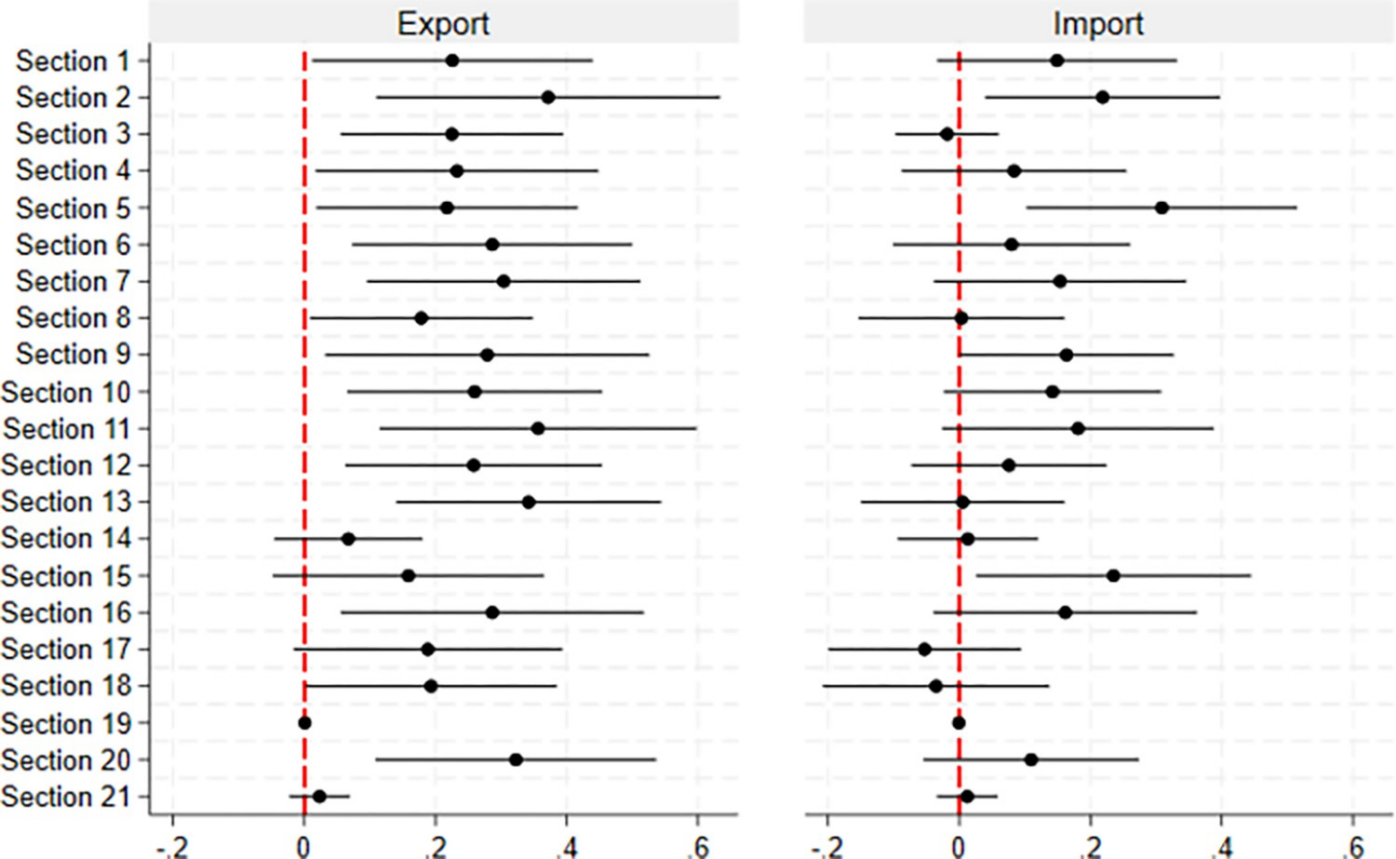
The impact of CKL on HS2 sections.

Lastly, we check which section of HS commodities is affected by CKL. Although we have shown CKL impacts the trade share between Chinese counties and South/North Korea, which specific commodity is affected by CKL remains unclear. To address this, we calculate the total export and import value (log) in different HS2 sections for each county, which consist of 21 classifications, to study. Fig 8 shows the results with the 95% confidence interval. For exports, only five sections are not significantly affected, suggesting that CKL can assist most domestic manufacturers in finding international buyers. On the import side, CKL affects only vegetable products, mineral products, and base metals. This implies that the impact of CKL on imports is more industry-specific. Thus, the beneficial role of CKL appears to be more general in terms of exports but more industry-specific when it comes to imports.

## 6. Conclusion

In this paper, we provide county-level evidence on the causal relationship between the common language and bilateral trade. We use the IV approach defended by the historical large-scale Korean migration, to address potential endogeneity issues. The results confirm that common language promotes bilateral trade. Specifically, for every 1% increase in the proportion of the Korean population, the trade value share and transactions share between North Korea and South Korea increase by 1.8% and 2.3%, respectively. It suggests a large elasticity of trade to CKL. These effects are more pronounced in trade with North Korea and in higher trade share regions. Furthermore, we show that the common Korean language exerts its influence through reducing communication barrier. The impact is mainly observed at the extensive margin rather than the intensive margin. These findings highlight the potential of leveraging minority languages to boost bilateral trade in developing countries.

This paper offers valuable insights for policymakers to implement policies regarding economic development from a linguistic and cultural perspective, particularly in developing countries. Given that a common language fosters bilateral trade, it is important to preserve and leverage language resources. Enhancing language communication with neighboring countries is crucial to promoting the growth of international trade.

While this study employs the IV method to explore the relationship between CKL and international trade, it is important to approach the findings with caution regarding claims of causality. The inability to control for county-level fixed effects introduces potential omitted variable issues that may influence the results. Factors such as distance, institutional frameworks, and cultural elements, which are time-invariant at the county level, play a significant role in shaping international trade patterns. These unobserved factors could potentially confound the estimated effects, thereby limiting the robustness of the causal inferences. Future research should aim to incorporate county-level fixed effects or other methodologies that can better isolate the impact of CKL on trade outcomes. Additionally, collecting more granular data over an extended period could help mitigate these limitations and provide a clearer understanding of the underlying influences.

## Supporting information

S1 Data (XLS)
